# Identification of oxeiptosis-associated lncRNAs and prognosis-related signature to predict the immune status in gastric cancer

**DOI:** 10.1097/MD.0000000000037189

**Published:** 2024-02-16

**Authors:** Li Wen, Kaili Xu, Min Huang, Qin Pan

**Affiliations:** a Nursing Department, The Second Affiliated Hospital of Zhejiang University School of Medicine, Hangzhou, China; b Department of Operating Room, The First People’s Hospital of Linping District, Hangzhou, China; c Department of Medicine, Zhejiang Rehabilitation Hospital, Hangzhou, China.

**Keywords:** gastric cancer, immune status, lncRNA, oxeiptosis, prognosis, risk signature

## Abstract

As a novel form of cell death, oxeiptosis is mainly caused by oxidative stress and has been defined to contribute to the cellular death program in cancer. However, the precise involvement of oxeiptosis-related long non-coding RNAs (lncRNAs) within gastric cancer (GC) remains elusive. Thus, our study was aimed to elucidate the pivotal effect of hub oxeiptosis-related lncRNAs on GC by comprehensively analyzing lncRNA and gene expression data obtained from The Cancer Genome Atlas (TCGA) database. Subsequently, we constructed a risk signature (risk-sig) using lncRNAs and further evaluated its prognostic significance. We successfully identified thirteen lncRNAs closely related with oxeiptosis that exhibited significant relevance to the prognosis of GC, forming the foundation of our meticulously constructed risk-sig. Notably, our clinical analyses unveiled a strong correlation between the risk-sig and crucial clinical parameters including overall survival (OS), gender, TNM stage, grade, M stage, and N stage among GC patients. Intriguingly, the diagnostic accuracy of this risk-sig surpassed that of conventional clinicopathological characteristics, underscoring its potential as a highly informative prognostic tool. In-depth mechanistic investigations further illuminated a robust association between this risk-sig and fundamental biological processes such as tumor stemness, immune cell infiltration, and immune subtypes. These findings provide valuable insights into the complex interplay between oxeiptosis-related lncRNAs and the intricate molecular landscape of GC. Ultimately, leveraging the risk scores derived from our comprehensive analysis, we successfully developed a nomogram that enables accurate prediction of GC prognosis. Collectively, our study established a solid foundation for the integration of thirteen hub oxeiptosis-related lncRNAs into a clinically applicable risk-sig, potentially revolutionizing prognostic assessment in GC and facilitating the development of innovative therapeutic strategies.

## 1. Introduction

Gastric cancer (GC) is the third leading cause of cancer-related mortality and the fifth most prevalent malignancy worldwide,^[1,2]^ posing a significant threat to public health. Approximately 95% of GC patients are diagnosed with malignant GC, which carries a poor prognosis.^[3]^ Due to limitations in early diagnosis and therapeutic methods, the 5-year survival rate for patients with advanced GC is <10%.^[4]^ Therefore, there is an urgent need to identify novel biomarkers and develop a sensitive prognostic signature to accurately predict the condition of patients with GC.

The cell death program encompasses 2 primary mechanisms: programmed cell death (PCD) as well as accidental cell death.^[5]^ Oxeiptosis, a caspase-independent cell death modality akin to apoptosis, has recently emerged as a novel type of PCD intricately linked to the excessive levels of reactive oxygen species (ROS).^[6]^ Substantial evidence supports the significant involvement of the KEAP1-PGAM5-AIFM1 pathway in the modulation of oxeiptosis.^[7]^ KEAP1 is a virtual sensor of ROS and ensures a sustained elevation in Nrf2 levels,^[8]^ subsequently augmenting the expression of multiple antioxidant-related genes to safeguard cells against moderate ROS stress.^[9]^ Under conditions of heightened intracellular ROS concentrations, oxeiptosis trigger PCD by harnessing the potential of KEAP1 to detect ROS.^[6]^ Under such circumstances, KEAP1 dissociates from PGAM5, facilitating the translocation of PGAM5 into the mitochondrial lumen, ultimately promoting AIFM1 dephosphorylation.^[10]^ Consequently, AIFM1 is transported to the nucleus, where it instigates DNA degradation, encompassing apoptosis and parthanatos, thereby culminating in chromatin condensation.^[11]^ Recent studies have indicated that oxeiptosis can influence the prognosis of patients with breast cancer.^[12]^ Nevertheless, its precise role in GC remains inadequately understood.

Long non-coding RNAs (lncRNAs), a subclass of non-coding RNAs, exert a pivotal role in the progression of tumors, encompassing tumor cell proliferation, tumorigenesis, as well as metastasis.^[13,14]^ Moreover, lncRNAs have been observed to exhibit strong correlations with the overall survival (OS) of individuals with cancer.^[15]^ Numerous experimental investigations, either in vitro or in vivo, have illustrated the regulatory influence of lncRNAs on various cellular processes in cancer cells, such as cell migration, invasion, apoptosis, as well as the progression of cell cycles. However, the potential association between lncRNAs implicated in oxeiptosis and the prognosis of GC remains largely unexplored. With the emergence alongside the implementation of bioinformatic analyses, numerous disease-specific biomarkers have been successfully identified. Nonetheless, up to now, no oxeiptosis-related lncRNAs along with the outcome or advancement of GC have been discovered. Hence, we employed univariate Cox regression as well as gene expression analyses to identify significant lncRNAs linked to GC patient prognosis, further exhibiting differential expression of normal individuals from that of GC patients. Following the identification of hub oxeiptosis-related lncRNAs, we proceeded to conduct a Least Absolute Shrinkage and Selection Operator (LASSO) penalized Cox regression analysis develop a prognostic risk signature (risk-sig). We further validated the clinical relevance as well as the prognostic value of this risk-sig using data from GC patients. Additionally, the associations of this risk-sig with tumor stemness (TS) as well as immune infiltration were explored. In summary, by constructing a risk-sig using lncRNAs related to oxeiptosis, we have created a useful instrument for forecasting the outcome of GC, providing new perspectives for its diagnosis.

## 2. Materials and methods

### 2.1. Acquisition of raw data

The RNA sequencing datasets for normal and GC tissues, specifically the The Cancer Genome Atlas (TCGA)-GC (comprising 375 GC samples as well as 32 normal samples), were obtained from reliable sources within TCGA (https://portal.gdc.cancer.gov/) database. All samples were derived from Homo sapiens. In accordance with previous studies,^[12]^ 5 genes related to oxeiptosis (PGAM5, KEAP1, AIFM1, NRF2, as well as AIRE) were selected and subjected to further analysis.

### 2.2. Construction of the lncRNA signature for prognosis

Upon conducting a rigorous analysis of the associations of GC and lncRNAs related with oxeiptosis via Pearson correlation analysis (|R^2^| > 0.2 & *P* < .05), we adopted the “limma” package within the R programming environment to systematically identify lncRNAs that were differentially expressed in the context of oxeiptosis. These candidate lncRNAs were stringently defined based on the criteria of false discovery rate  < 0.05 and |log2 fold change | > 1 when comparing tumorous and adjacent normal tissues. Subsequent analytical steps involved the application of the “survival” package in R, wherein univariate Cox proportional hazards regression analysis was meticulously performed to discern prognostic lncRNAs related with oxeiptosis from the entirety of the lncRNA dataset, adopting a rigorous *P* value cutoff of <.001. Through this integrative approach, we delineated a subset of lncRNAs that were both prognostically significant and differentially expressed, thereby identifying them as our prime candidate lncRNAs implicated in oxeiptosis. To visually represent the intersecting sets of lncRNAs, we employed the “VennDiagram” package to construct a Venn diagram, thereby facilitating a clear graphical elucidation of our findings.

Subsequently, to identify the hub lncRNA and establish a robust risk-sig, the selected lncRNAs were integrated through a Lasso penalized Cox regression analysis. The risk score (RS) of risk-sig generated from the hub oxeiptosis-related lncRNAs was meticulously formulated according to the following equation:


$$\begin{array}{l} RS=\Sigma explnci*\beta i \end{array}$$

where explnci symbolizes the relative expression of the hub lncRNA related to oxeiptosis, while β corresponds to the regression coefficient attributed to each lncRNA, as determined by the Lasso regression model.

Based on the value of the RS, patients diagnosed with GC were catrgorized into 2 distinct prognostic subgroups: low - risk (LR)/high -risk (HR) subgroups.

### 2.3. Predictive value of the lncRNA signature

To investigate the distribution of LR and HR subgroups, we utilized the packages of “ggplot2” as well as “Rtsne,” conducting PCA and t-SNE. We compared the prognostic ability based on the levels of the RS utilizing Cox regression alongside survival analyses. Subsequently, we employed the “timeROC” package to determine the predictive accuracy of this risk-sig. A nomogram was developed utilizing the “rms” package to predict the outcomes of GC patients based on RSs. Additionally, we conducted a decision curve analysis to assess the accuracy as well as the discrimination.

### 2.4. Gene set enrichment analysis (GSEA)

To analyze the enrichment of these oxeiptosis-related lncRNAs, GSEA 4.1 was applied, and the KEGG enrichment analysis was carried out. An false discovery rate < 0.05 was set as statistically significant.

### 2.5. Immune and stem cell-like features correlation analysis

To investigate immune functionality and assess the differential immune cell infiltration of the subgroups, we performed a single-sample GSEA (ssGSEA). The relationship between the RS and immune or stromal factors was assessed through Spearman correlation analysis. A 2-way analysis of variance was used to scrutinize the association between the RS and the subtypes of immune cell infiltration. Moreover, the connection of the risk-sig and immune-related genes was elucidated by examining potential immune checkpoints that were identified in an earlier research.^[16]^ Subsequently, we rigorously evaluated the putative associations between the risk-sig with the expression of Programmed Death-Ligand 2 (PD-L2). Additionally, we assessed the relationship between TS and the RS using Spearman correlation analyses.

## 3. Results

### 3.1. Screening of prognostic lncRNA candidates

**Figure 1.**
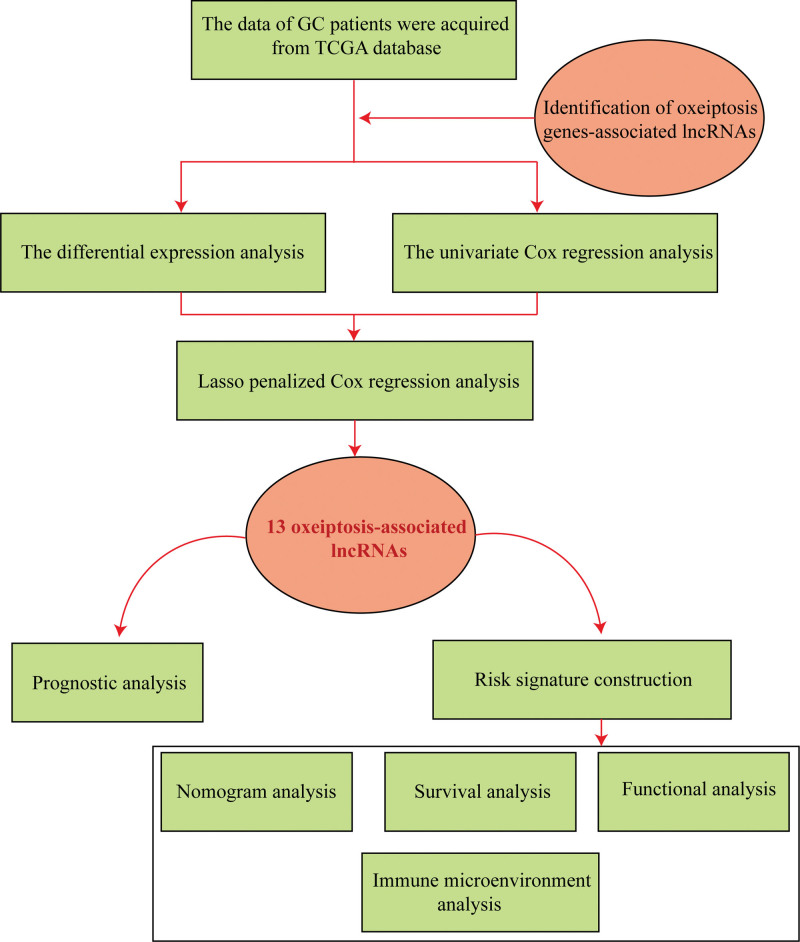
Schema of the study.

**Figure 2.**
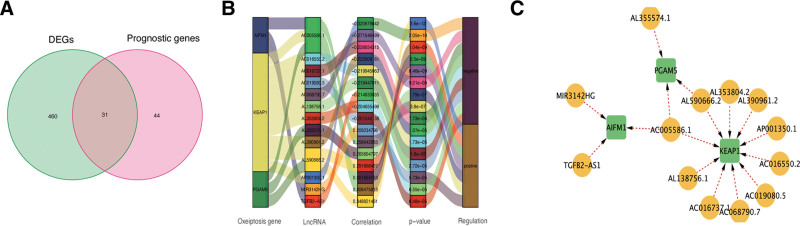
Identification of prognostic oxeiptosis-associated lncRNAs. (A) Venn diagram of candidate oxeiptosis-associated lncRNAs determined by differential expression and univariate Cox analyses. (B) Correlation network of prognostic lncRNAs and their associated mRNAs. (C) Correlation network of hub lncRNAs. lncRNAs = long non-coding RNAs.

The study workflow is depicted in Figure 1. Correlation analysis based on the criteria of |R2| > 0.2 and *P* < .05 identified 1211 lncRNAs related with oxeiptosis with statistical significance (Supplementary Table 1, http://links.lww.com/MD/L641). These lncRNAs were then applied for further explorations. Besides, our study identified 491 differentially expressed lncRNAs (Supplementary Tables 2, http://links.lww.com/MD/L642) and 75 lncRNAs related with the prognosis of GC (Supplementary Tables 3, http://links.lww.com/MD/L643) based on the methods of differential expression analysis alongside univariate Cox regression. At last, a group of 31 overlapping lncRNAs were found and adopted as potential candidates for in-depth prognostic analysis, as shown in Figure 2A.

### 3.2. Construction of the risk signature

Based on the above candidates, Lasso penalized Cox regression analysis was then performed and a risk-sig consisted of 13 hub lncRNAs were constructed, involving AP001350.1, AC019080.5, AC068790.7, AC016737.1, MIR3142HG, AL138756.1, TGFB2-AS1, AL353804.2, AL390961.2, AL355574.1, AL590666.2, AC016550.2, as well as AC005586.1 (Supplementary Table 4, http://links.lww.com/MD/L644). Figure 2B and C show the connections of these hub lncRNAs and the genes related with oxeiptosis.

### 3.3. Analysis of the prognostic value of oxeiptosis-related lncRNAs in GC

**Figure 3.**
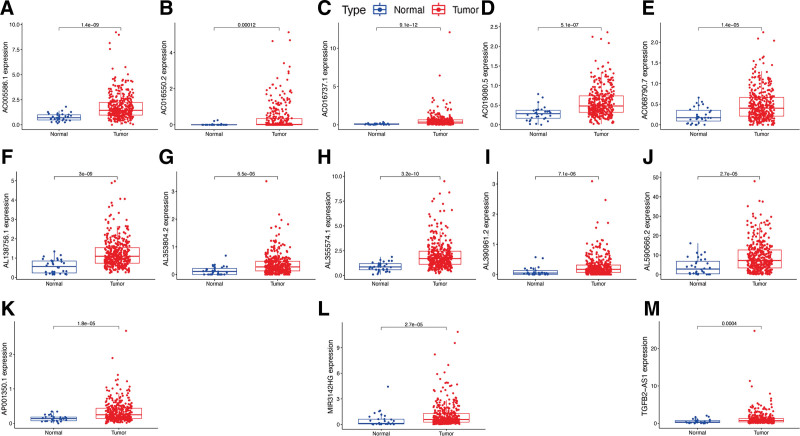
Gene expression of hub lncRNAs in GC. Gene expression levels of AC005586.1 (A), AC016550.2 (B), AC016737.1 (C), AC019080.5 (D), AC068790.7 (E), AL138756.1 (F), AL353804.2 (G), AL355574.1 (H), AL390961.2 (I), AL590666.2 (J), AP001350.1 (K), MIR3142HG (L), and TGFB2-AS1 (M) in GC and normal samples. GC = gastric cancer, lncRNAs = long non-coding RNAs.

**Figure 4.**
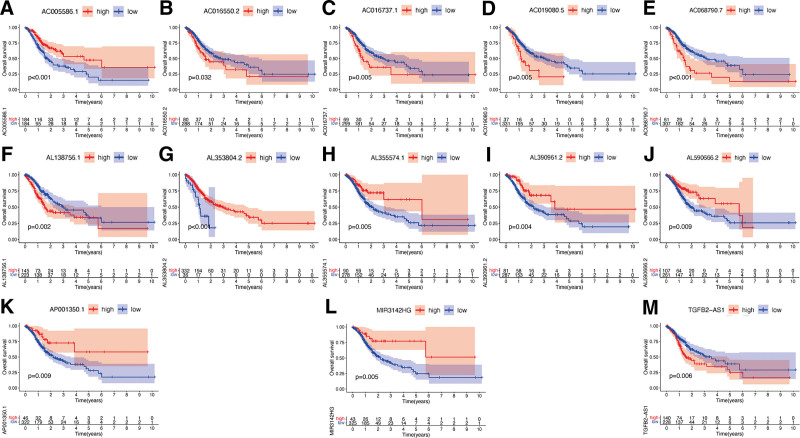
Survival curve of hub lncRNAs in GC. Survival curve of AC005586.1 (A), AC016550.2 (B), AC016737.1 (C), AC019080.5 (D), AC068790.7 (E), AL138756.1 (F), AL353804.2 (G), AL355574.1 (H), AL390961.2 (I), AL590666.2 (J), AP001350.1 (K), MIR3142HG (L), and TGFB2-AS1 (M) in GC. GC = gastric cancer, lncRNAs = long non-coding RNAs.

All hub lncRNAs, including AC005586.1 (Fig. 3A), AC016550.2 (Fig. 3B), AC016737.1 (Fig. 3C), AC019080.5 (Fig. 3D), AC068790.7 (Fig. 3E), AL138756.1 (Fig. 3F), AL353804.2 (Fig. 3G), AL355574.1 (Fig. 3H), AL390961.2 (Fig. 3I), AL590666.2 (Fig. 3J), AP001350.1 (Fig. 3K), MIR3142HG (Fig. 3L), and TGFB2-AS1 (Fig. 3M), all showed higher expression levels in GC tissues with statistical significance (*P* < .05). Besides, results of KM survival analysis indicated that the subgroups of GC patients with the higher AC005586.1 (Fig. 4A), AL353804.2 (Fig. 4G), AL355574.1 (Fig. 4H), AL390961.2 (Fig. 4I), AL590666.2 (Fig. 4J), AP001350.1 (Fig. 4K), and MIR3142HG (Fig. 4L) expression levels had a better OS with statistical significance (*P* < .05). Additionally, elevated expression levels of AC016550.2 (Fig. 4B), AC016737.1 (Fig. 4C), AC019080.5 (Fig. 4D), AC068790.7 (Fig. 4E), AL138756.1 (Fig. 4F), and TGFB2-AS1 (Fig. 4M) corresponded to worse outcomes of GC patients with statistical significance (*P* < .05).

### 3.4. Value of risk signature in clinics

**Figure 5.**
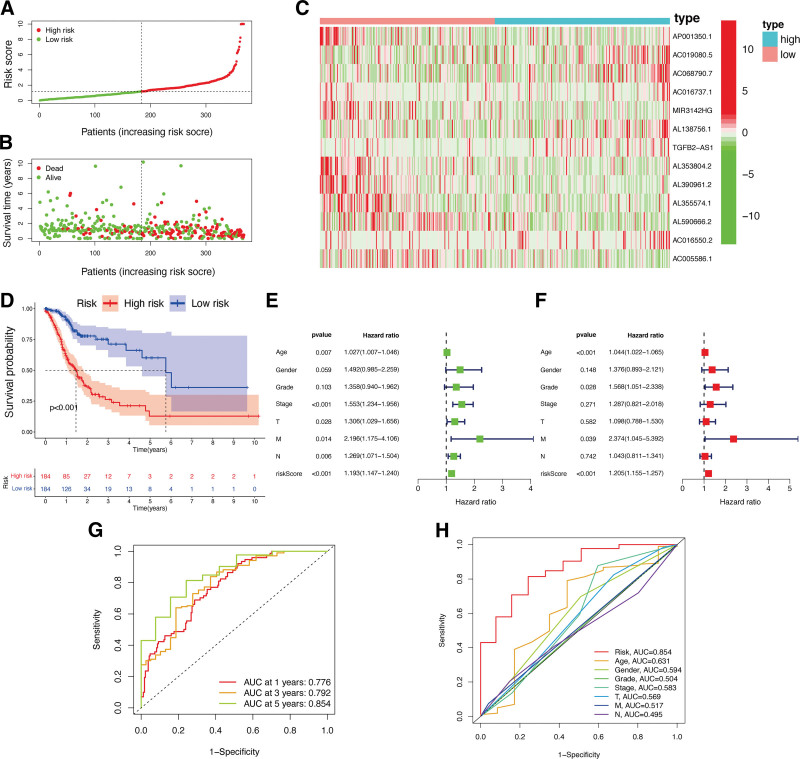
Associations between risk signature and GC prognosis. Risk score distribution (A) and survival status (B) analysis of TCGA-GC cohort. (C) Expression level of hub lncRNAs in risk subgroups. (D) Survival curve of GC patients. Univariate (E) and multivariate Cox (F) regression of clinicopathological features. TimeROC (G) and ClinicalROC (H) curves to forecast overall survival of patients. GC = gastric cancer, lncRNAs = long non-coding RNAs, TCGA = The Cancer Genome Atlas.

According to the median RS, patients with GC were categorized into LR and HR subgroups with low and high RSs (Fig. 5A and B). Assessment of lncRNA expression in these subgroups (Fig. 5C) revealed that AC019080.5, AC068790.7, AL138756.1, TGFB2-AS1, and AC016550.2 were upregulated in the HR subgroup with statistical significance (*P* < .05). Conversely, in contrast with to the LR subgroup, HR subgroup had lower expression levels of AP001350.1, AC016737.1, MIR3142HG, AL353804.2, AL390961.2, AL355574.1, AL590666.2, and AC005586.1 with statistical significance (*P* < .05). Besides, in contrast with patients with low-RSs, those with high-RSs exhibited a lower OS (*P* < .0), as shown in Figure 5D. The univariate Cox regression found that the risk-sig was related to GC patients’ OS with statistical significance (Fig. 5E). Furthermore, our analysis demonstrated this risk-sig to be an independent prognostic factor for GC patients, as shown in Figure 5F. ROC curve analysis demonstrated its moderate predictive accuracy, with the AUC value of 0.776, 0.792, and 0.854 for 1, 3, and 5 years, respectively (Fig. 5G). Notably, when compared with traditional clinicopathological features, the decision and ROC curve analyses (Fig. 5H) indicated higher accuracy for this risk-sig, emphasizing its sensitivity and specificity for predicting OS in GC.

**Figure 6.**
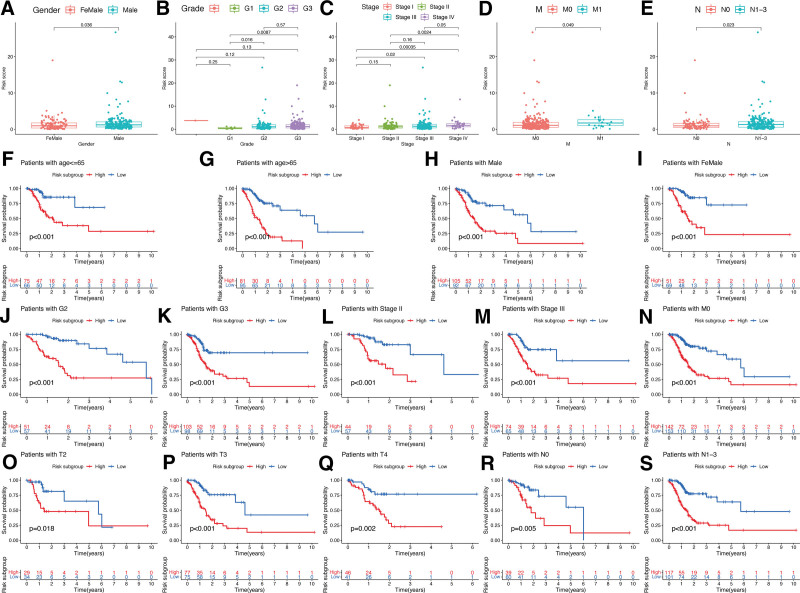
Associations between risk signature and clinicopathological factors. Correlations between risk scores and gender (A), cancer grade (B), TNM stage (C), M stage (D), and N stage (E). The prognosis of risk signature under the stratifications of (F, G) age ≤ 65 and age > 65; (H, I) male and female; (J, K) G2 and G3; (L, M) TNM stage II and III; (N) M0 stage; (O-Q) T2, T3, and T4 stage; (R, S) N0 and N1-3 stage.

Moreover, compared to the females, the males showed higher RSs with statistical significance (*P* < .05; Fig. 6A). Similarly, RSs were also higher in patients at G2/G3 stage compared to those at G1 stage (*P* < .05; Fig. 6B). Substantially higher RSs were also observed in patients with advanced TNM stage, M1 stage, or N1-3 stage compared to those with earlier stages (*P* < .05; Fig. 6C–E). Additionally, the prognostic value of the risk-sig was examined across various clinical characteristics. For patients categorized by age, gender, tumor grade, TNM stage, and metastasis status, critically different OS were observed between LR- and HR subgroups (Fig. 6F–S). In all these cases, patients characterized by HR signatures demonstrated a considerable OS disadvantage when compared to those with LR signatures.

**Figure 7.**
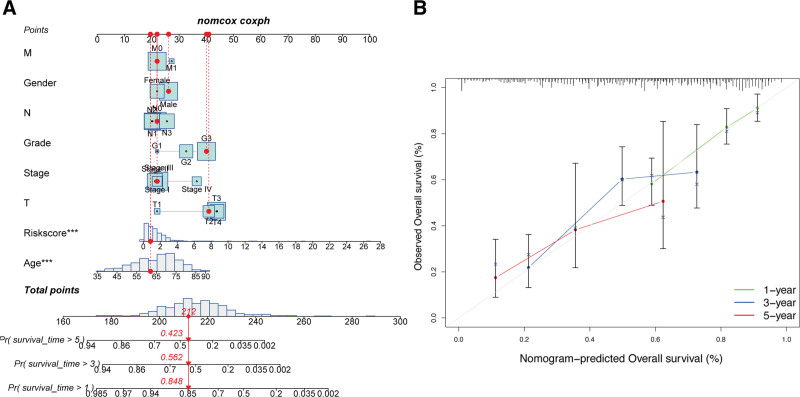
Construction of nomogram. (A) Nomogram for predicting GC 1-, 3-, and 5-yr OS in TCGA cohort. The red dashed line represented a sample of GC patient death probability by year 1, 3, and 5. (B) Decision curve analysis of risk signature and other clinicopathological features. GC = gastric cancer, OS = overall survival, TCGA = The Cancer Genome Atlas.

Subsequently, a nomogram incorporating the identified risk signatures was constructed to predict outcomes for GC patients (Fig. 7A), and calibration curves demonstrated substantial agreement across 1-, 3-, and 5-year follow-up (Fig. 7B). In view of its close association with the development of GC, the nomogram with established risk-sig may stand as a useful approach for the management of patients with GC in clinical settings.

### 3.5. GSEA

**Figure 8.**
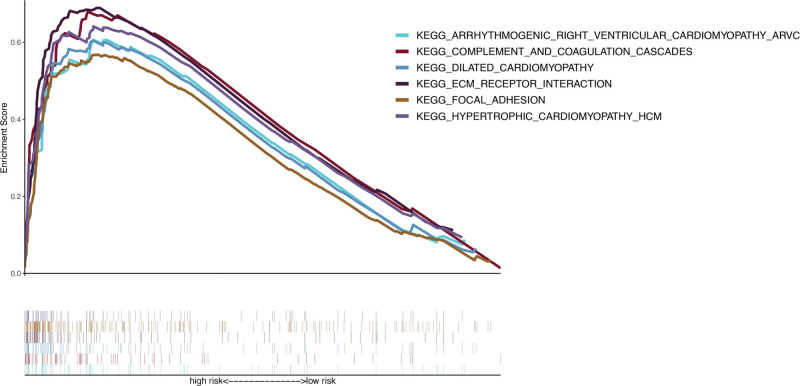
GSEA of enriched pathways in risk signature. GSEA = gene set enrichment analysis.

Using GSEA, significant enrichments of the lncRNA signature were identified in pathways such as arrhythmogenic right ventricular cardiomyopathy ARVC, complement and coagulation cascades, dilated cardiomyopathy, EGC receptor interaction, focal adhesion, and hypertrophic cardiomyopathy HGC (Fig. 8).

### 3.6. Relationship of the risk signature with immunity and TS

**Figure 9.**
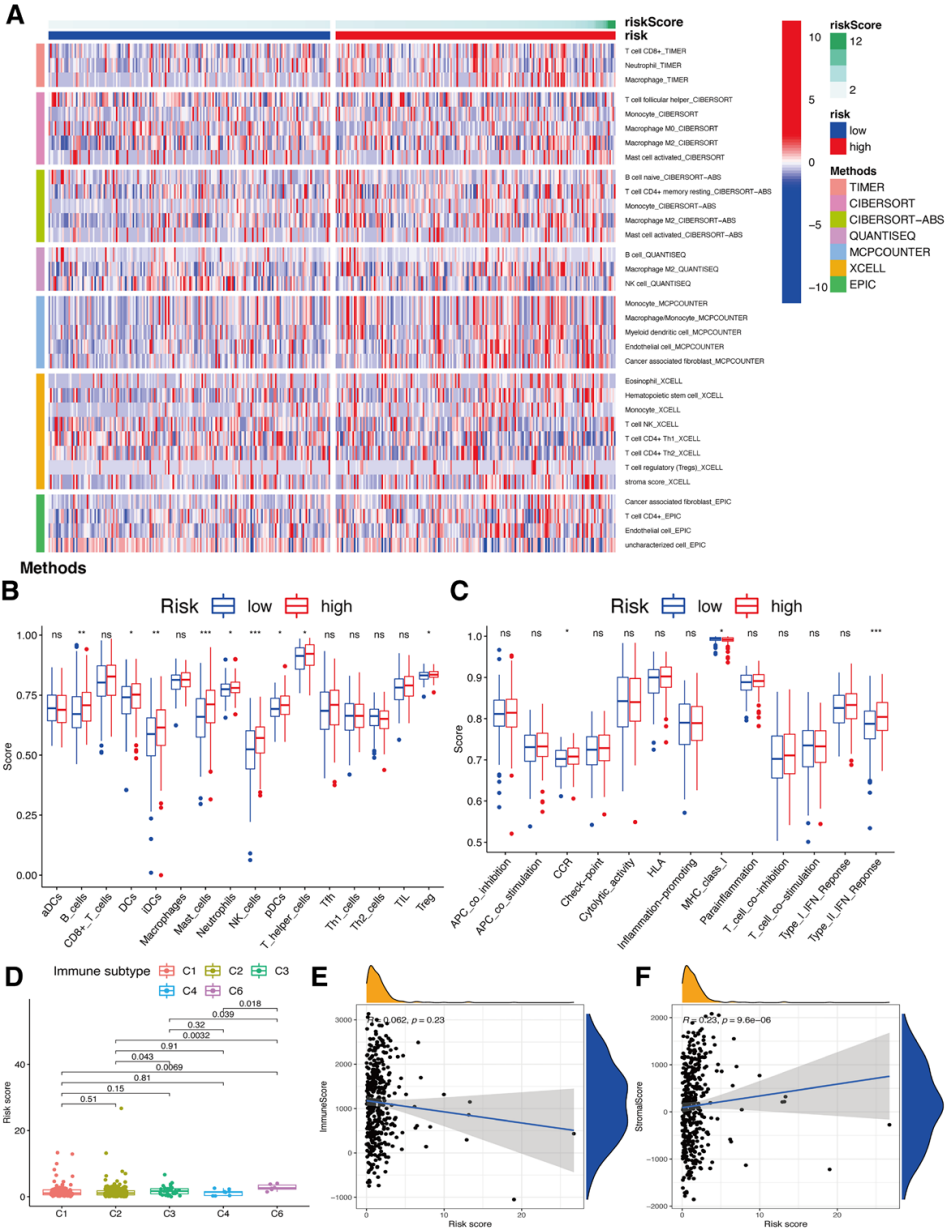
Potential role of risk signature in GC immune status. (A) Heatmap for immune responses based on EPIC, XCELL, MCP counter, QUANTISEQ, CIBERSORT, and TIMER among 2 risk subgroups. Boxplots of scores of immune cells (B) and immune-associated functions (C) in risk subgroups. Associations between risk signature and immune infiltration subtypes (D), immune scores (E), and stromal scores (F). GC = gastric cancer.

Based on analyses from TIMER, CIBERSORT, QUANTISEQ, MCP counter, XCELL, as well as EPIC, we found the risk-sig was closely related with certain immune cell types (Fig. 9A). Patients characterized by high RSs demonstrated a noteworthy increase in diverse immune cell subpopulations and their corresponding functions, such as B cells, dendritic cells (DCs), immature dendritic cells (iDCs), mast cells, neutrophils, natural killer (NK) cells, pDCs, helper T cells, Treg, CCR, class I major histocompatibility complex (MHC), as well as Type II immune interferon (IFN) response, when compared to those with low RSs (Fig. 9B and C) (*P* < .05). Besides, we explored the connection of the risk-sig and immune infiltrates related to the promotion and the suppression of tumors,^[17]^ such as wound healing (C1), INF-g dominant (C2), inflammatory (C3), lymphocyte-depleted (C4), immunologically quiet (C5), as well as transforming growth factor (TGF)-beta dominant (C6) subtypes. Notably, we found a significantly higher RS related with the C6 subtype (Fig. 9D). Additionally, we examined the linkage between the risk-sig and the immune microenvironment, specifically stromal and immune scores. Our findings indicated a significant positive correlation between the established risk-sig and stromal scores (*P* < .05; Fig. 9F), however, no significant connection was observed between the RS and immune score (*P* > .05; Fig. 9E).

**Figure 10.**
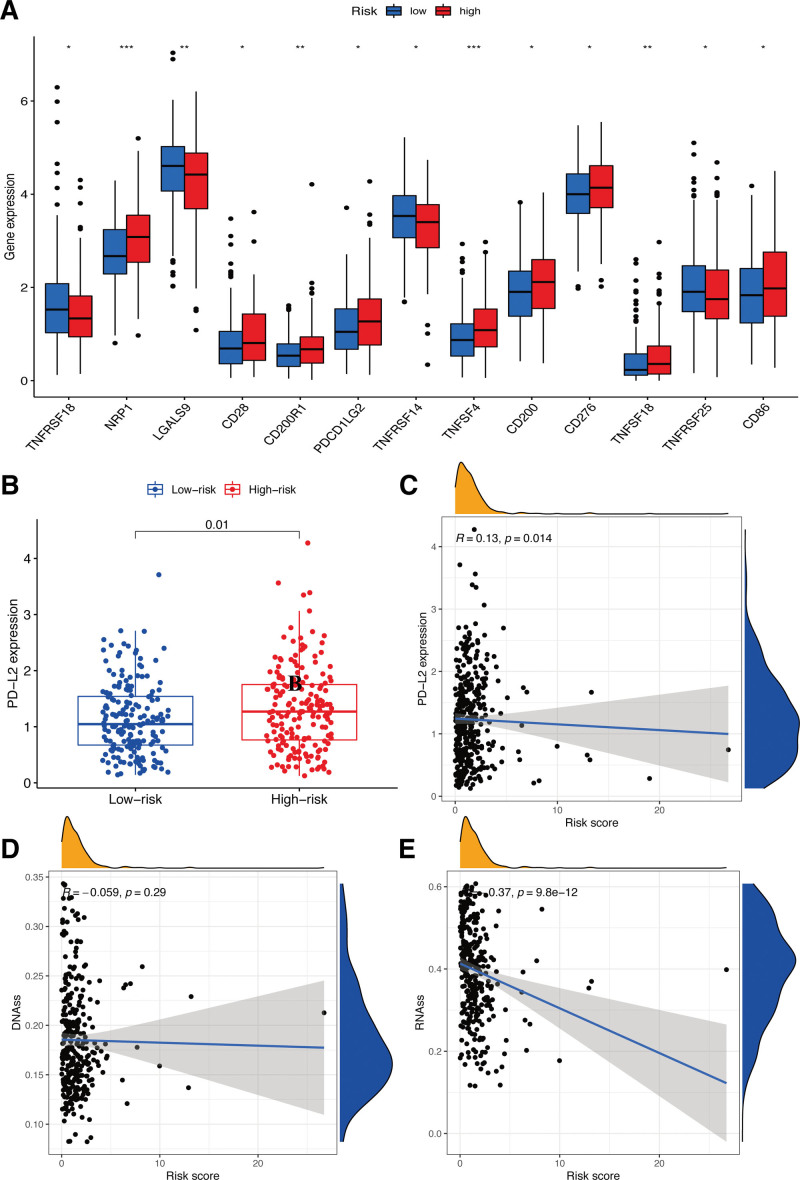
Potential role of risk signature in immune checkpoints and tumor stemness. (A) Expression of immune checkpoints among 2 risk subgroups in GC patients. Expression levels of genes PD-L2 (B) in risk subgroups. Correlation analysis between risk score and PD-L2 (C). Associations between risk signature and DNAss (D) and RNAss (E). GC = gastric cancer.

In terms of immune checkpoints, several differentially expressed genes in relation with the immunity were observed within the subgroups (*P* < .05), as shown in Figure 10A. The subgroup with high RSs demonstrated notably higher expression of most genes, including NRP1, CD28, CD200R1, PDCD1LG2, TNFSF4, CD200, CD276, TNFSF18, and CD86, except for TNFRSF18, LGALS9, TNFRSF14, and TNFRSF25. Additionally, we extensively explored the association between PD-L2 loci and the risk-sig, revealing significantly increased PD-L2 expression in patients with high RSs in contrast with those with low RSs (Fig. 10B). Furthermore, we identified an evident negative correlation between PD-L2 expression and the calculated RS (Fig. 10C).

Considering TS, including DNA methylation pattern together with RNA stemness score (RNAss), we found a negative correlation of the risk-sig and RNAss (*P* < .05); However, no such correlation was found between the risk-sig and the patterns of DNA methylation (DNAss; *P* > .05) (Fig. 10D and E).

## 4. Discussion

With the advancement of next-generation sequencing (NGS) technology in the field of biological research, numerous biomarkers for GC have been discovered. Despite of that, there is an urgent need for biomarkers that can aid in early detection and prognostic prediction for this condition. Oxeiptosis, an emerging cell death mechanism, is vital for cancer.^[12]^ Nevertheless, its involvement in cancer generation, development, progression, and metastasis remains unclear, and little is known about lncRNA signatures related with oxeiptosis. In this study, we established a new risk-sig and confirmed its predictive accuracy of OS for GC patients. Additionally, we observed substantial correlations among this risk-sig, TS, immune components, tumor microenvironment, as well as immune status, indicating its potential advantages.

To ascertain the connections between lncRNAs and the OS among patients with GC, we conducted a comprehensive analysis of genes related with oxeiptosis, namely PGAM5, KEAP1, AIFM1, NRF2, as well as AIRE. Next, to establish a prognostic risk-sig for GC patients, we employed 13 hub lncRNAs: AP001350.1, AC019080.5, AC068790.7, AC016737.1, MIR3142HG, AL138756.1, TGFB2-AS1, AL353804.2, AL390961.2, AL355574.1, AL590666.2, AC016550.2, and AC005586.1. We utilized various approaches to confirm the value of this prognostic risk-sig for GC patients. Our findings revealed a strong link of this risk-sig to tumor TNM stage, patient gender, tumor grade, M stage, and N stage. As a commonly used clinicopathological parameter, the American Joint Committee on Cancer staging system is adopted for evaluating tumors.^[18]^ In contrast with the TNM stage, the risk-sig demonstrated high accuracy in predicting the growth, metastasis, and prognosis of GC. Nomogram analysis further confirmed the effectiveness of this prognostic risk-sig for GC patients.

Moreover, the associations of this risk-sig and immune-related cells were confirmed using several methods, such as TIMER, CIBERSORT, QUANTISEQ, MCPCOUNTER, XCELL, as well as EPIC. These associations, along with immune processes, signify the prognostic value of this risk-sig. Notably, in patients with high RSs, almost all immune cells demonstrated higher infiltration as well as impaired immune functions. Based on the pivotal effects of immune cells on anti-tumor immunity,^[19]^ it could be speculated that anti-tumor immune responses are markedly activated among GC patients with high RSs. Furthermore, we observed significant positive correlations between the RSs and stromal cell scores, implying higher immune cell infiltration in GC patients with high RSs. Additionally, we found a strong association of increased RSs with the C6 subtype, suggesting the predictive value of the RS for OS as well as its protective effect for GC.

At present, immunotherapies targeting immune checkpoints are reported to be highly effective for improving cancer outcomes.^[20]^ The therapeutic success of immunotherapies is predominantly contingent upon the interaction of PD-L1 and PD-L2 with cancer.^[21]^ Studies have shown that antibodies targeting PD-1/PD-L1 can yield substantial treatment outcomes by interrupting the inhibitory mechanism mediated by PD-L1 and thereby augmenting the functional capacity of T-cells.^[22,23]^ In the present study, we verified PD-L1 and PD-L2 expressions across different subgroups and observed that PD-L2 expressions were negatively related with RSs. Furthermore, patients with the high RS showed markedly higher expression of most immune checkpoint molecules compared to those with low RSs, indicating notable alterations in immune responses within the HR group. Consequently, the risk-sig established in this study may have a predictive effect on the expressions of immune checkpoints among GC patients, thereby potentially guiding GC immunotherapy. Despite of that, further exploration is necessary to analyze the link of lncRNAs related with oxeiptosis and immune-related genes.

Cancer stem cell-like cells can obviously influence the growth as well as the progression of tumors based on their invasive and self-renewal activities. Additionally, the resistance of tumor cells to chemotherapy drugs is notably related with cancer stem cell-like cells.^[24,25]^ In this context, the lncRNA signature was in a negative association with RNAss, indicating its potential as a risk factor of GC.

While this study identified hub oxeiptosis-related lncRNAs within the context of GC and introduced a prognostic risk-sig demonstrating robust predictive power for patient outcomes. Nevertheless, the study had several limitations. Firstly, the gene expression profiles as well as clinical data pertaining to GC were extracted exclusively from publicly accessible repositories, thereby necessitating empirical validation via supplementary experimental methodologies. Secondly, prospective studies should be conducted to substantiate the findings elicited from the present study. Furthermore, comprehensive functional/mechanistic explorations maybe imperative to elucidate the intricate roles as well as the underlying mechanisms by which oxeiptosis-related lncRNAs influence the pathogenesis of GC.

## 5. Conclusions

The present study elucidated the pivotal effect of hub lncRNAs related with oxeiptosis and established a new risk-sig for the stratification of GC patients. The identified lncRNAs not only enhanced the prognostic accuracy concerning OS in GC but also served as surrogate markers for immune conditions of patients. Using the lncRNAs related with oxeiptosis within the realm of oncology, we generated the first risk-sig, which could be applied in the novel therapeutic strategies of GC treatment.

## Author contributions

**Conceptualization:** Kaili Xu.

**Data curation:** Kaili Xu.

**Formal analysis:** Kaili Xu.

**Investigation:** Kaili Xu.

**Methodology:** Min Huang.

**Project administration:** Kaili Xu.

**Software:** Min Huang.

**Validation:** Qin Pan.

**Visualization:** Qin Pan.

**Writing – original draft:** Kaili Xu.

**Writing – review & editing:** Li Wen, Qin Pan.

## Supplementary Material








